# Toward a resilient opioid treatment workforce: Clinical supervision, training, and trauma-related functioning in a multistate study

**DOI:** 10.1080/1533256x.2026.2703982

**Published:** 2026-07-27

**Authors:** Linnea B. Linde-Krieger, Elena Tennant, Alissa Davis, Benjamin R. Brady, Patrick S. Rivers, Gregory A. Carter, Zhanette H. Coffee, David Frank, Arelene N. Mahoney, Sara M. Najar, Richard A. Crosby, Beth E. Meyerson

**Affiliations:** a School of Social Work, Arizona State University, Phoenix, AZ, USA;; b Harm Reduction Research Lab, College of Nursing, University of Arizona, Tucson, AZ, USA;; c Comprehensive Center for Pain and Addiction, University of Arizona, Tucson, AZ, USA;; d School of Social Work, Columbia University, New York, NY, USA;; e College of Health and Human Services, Western Michigan University, Kalamazoo, MI, USA;; f College of Education and Human Development, Western Michigan University, Kalamazoo, MI, USA;; g College of Medicine-Tucson, University of Arizona, Tucson, AZ, USA;; h School of Nursing, Indiana University, Bloomington, IN, USA;; i School of Global Health, New York University, New York, NY, USA;; j Southwest Recovery Alliance, Phoenix, AZ, USA;; k College of Public Health, University of Kentucky, Lexington, KY, USA

**Keywords:** Opioid treatment programs, Clinical supervision, Vicarious trauma, Burnout, Reflective supervision, Substance use disorder treatment workforce, Secondary traumatic stress

## Abstract

Staff in opioid treatment programs (OTPs) face elevated risk of secondary traumatic stress, yet the availability and effectiveness of training and clinical supervision to address this risk remain unclear. This study characterized current OTP training and supervision practices and examined their associations with staff vicarious trauma (VT) and burn-out. In a cross-sectional survey of OTPs, administrators (*N* = 44, 21 U.S. states) reported on staff training. Staff (*N* = 83) completed validated measures of VT and burnout. Counselors (*n* = 30) reported on clinical supervision. Training in VT and burnout was widely available, and most administrators (83.3%) and staff (89.6%) expressed interest in reflective supervision tailored to OTPs. In regression models, clinical supervision was associated with lower VT and burnout, whereas training availability was associated with higher VT and burnout. Findings suggest existing training models may be insufficient to address OTP staff stress. Expanding relationally-focused supervision may buffer the emotional toll of substance use treatment work.

Social workers, addiction counselors, and other frontline clinical staff in substance use disorder (SUD) treatment settings face elevated risk of secondary traumatic stress, burnout, and job turnover (Bride & Kintzle, 2011; Murphy & Kruis, 2023). In their daily work, these providers care for clients navigating complex trauma histories, systemic stigma, and cumulative life stressors that contribute to repeated treatment disruptions (Bøhle et al., 2023; Odenwald & Semrau, 2013). The impact of sustained exposure to client trauma is further intensified by structural constraints, including strict federal and state regulatory requirements, workforce shortages alongside high patient volumes, and limited access to effective, evidence-based treatment options (Kelly et al., 2026). Systemic and regulatory challenges are especially pronounced in opioid treatment programs (OTP), which are the only U.S. treatment settings federally authorized to dispense methadone for opioid use disorder (Substance Abuse and Mental Health Services Administration, 2024). Prior studies by our research group and others show that high prevalence of client and staff trauma exposure and limited organizational resources leave OTP providers vulnerable to vicarious trauma (VT) reactions and burnout (Beitel et al., 2018; Coffee et al., 2025; Linde-Krieger et al., 2025). In turn, these factors contribute to staff turnover that undermines workforce stability and quality of care (Kelly et al., 2026). Despite the multiple challenges facing OTP providers, few evidence-informed models exist to support the emotional and professional development needs of this workforce.

Continuing education training and clinical supervision (i.e., a formal, structured, and collaborative relationship between an advanced clinician/supervisor and supervisee) are common professional development resources for social workers, counselors, and other addiction treatment providers (Aristy, 2025; El Amanni & Bartley, 2025), yet little is known about the availability, effectiveness, or content of these services in OTP settings. Extant studies have not examined whether training and clinical supervision are effective strategies to mitigate OTP provider VT and burnout. Limited evidence from other care fields suggests that continuing education may support enhanced staff well-being, particularly when trainings contain evidence-based stress management skills and extend beyond a single session (Garcia et al., 2026; Workman et al., 2026). A larger body of research documents clinical supervision as an effective strategy to mitigate provider stress and burnout, though the type and format of supervision and the quality of the supervisory relationship may modify these protective effects (Hiebler-Ragger et al., 2021; Sellers et al., 2025). In particular, *reflective supervision*, a relational and trauma-informed professional development approach, has been shown to foster provider resilience, reduce burnout, and enhance service quality (Eaves et al., 2022; Frosch et al., 2018; Morelen et al., 2022), but it has not been systematically adopted in substance use treatment contexts, including OTPs. To explore this, we surveyed a national sample of OTP administrators and staff to characterize training and clinical supervision practices in OTPs, measure their associations with staff VT symptoms and burnout, and assess interest and likelihood of adoption of a reflective supervision program tailored to the unique needs of OTPs.

## Vicarious trauma and burnout among OTP providers

Although SUD treatment providers work with clients with extensive trauma histories (Bride & Kintzle, 2011), they are unlikely to receive the specialized training and supervision commonly available in trauma-based service settings (Bride et al., 2009; Ewer et al., 2015). When providers are ill-prepared or unsupported in their work with trauma-impacted populations, risk for secondary trauma reactions increases (Newell & MacNeil, 2010). Vicarious trauma (VT) is a serious form of occupational stress that results from ongoing engagement with secondary traumatic material (e.g., repeatedly hearing about client traumatic experiences, witnessing client trauma responses; McCann & Pearlman, 1990). VT is characterized by emotional distress, changes in cognition and worldview (e.g., developing a belief that the world is a dangerous place), and physiological and psychological reactions that can mirror posttraumatic stress disorder (Canfield, 2005; Cummings et al., 2021; Pearlman & Saakvitne, 1995). Among SUD treatment providers, clinically significant VT symptom prevalence ranges from 20% to 50% depending on the population and setting (Ewer et al., 2015; Klingemann et al., 2024; Mavridoglou et al., 2026). Recent research among OTP staff (Linde-Krieger et al., 2025) documents elevated levels of VT exposure (>80%) and symptoms (>70%) that equal or exceed those observed in other high-risk professions (e.g., emergency room physicians, first responders, sexual assault clinicians). In addition, many OTP providers carry their own personal histories of trauma and posttraumatic stress symptoms, which can amplify the emotional toll of their work with trauma-impacted clients (Baird & Kracen, 2006; Leung et al., 2023). These cumulative stressors can negatively affect provider functioning and performance, contributing to emotional exhaustion and reduced capacity for therapeutic engagement (Canfield, 2005; Guitar & Molinaro, 2017; Showalter, 2010).

Research demonstrates that burnout, a syndrome marked by provider emotional exhaustion, cynicism, and reduced efficacy (Edú-Valsania et al., 2022; Maslach et al., 2001), is also prevalent in OTP settings (Beitel et al., 2018; Coffee et al., 2025; Murphy & Kruis, 2023). Burnout is consistently linked with VT symptomatology and turnover intentions (Cummings et al., 2021; Gkremou et al., 2026; Klingemann et al., 2024). Studies of addiction professionals find that 20%−60% experience moderate to high levels of burnout (Ciesluk et al., 2026; Coffee et al., 2025; Fentem et al., 2023; Klingemann et al., 2024; Volker et al., 2010). In conjunction with VT, burnout is exacerbated by systemic factors such as unmanageable caseloads, administrative burdens, and lack of organizational support (Kelly et al., 2026; Yang & Hayes, 2020). In substance use treatment programs, including OTPs, these phenomena frequently overlap. Left unaddressed, they contribute to fragmented care and poor therapeutic alliance (Knudsen et al., 2008; Oser et al., 2013; Raunick et al., 2015; Showalter, 2010). Despite these risks, emotional support for staff is often limited to administrative, compliance-driven supervision or informal peer mentoring (Bride et al., 2009; Ewer et al., 2015). An absence of structured, trauma-informed support mechanisms leaves providers vulnerable to disengagement and diminished performance, and, ultimately, leads to attrition of experienced and compassionate providers from the addiction treatment field (Guitar & Molinaro, 2017; Knudsen et al., 2008; Murphy & Kruis, 2023; Yang & Hayes, 2020).

## Evidence of training and clinical supervision effects on provider well-being

Emerging evidence suggests that continuing education can contribute to improvements in provider well-being, particularly when trainings incorporate active and evidence-based skill-building components such as stress management or self-compassion (Mösler et al., 2023). Interventions that extend beyond a single session and include opportunities for practice and reinforcement appear to be more effective than passive or standalone trainings (Garcia et al., 2026; Workman et al., 2026). For example, structured programs integrating resilience-building strategies have been associated with short-term reductions in emotional exhaustion among healthcare workers (Sexton & Adair, 2024). Targeted stress reduction training interventions, including mindfulness-based approaches and professional coaching, may reduce burnout and improve job satisfaction, though effect sizes and whether improvements are sustained over time vary across studies (Collett et al., 2026; Eriksson et al., 2018; Giordano et al., 2025; Mösler et al., 2023). Trainings that enhance providers’ sense of competence and self-efficacy, such as those focused on trauma-informed and evidence-based practices, can reduce provider turnover intentions and may be particularly beneficial in high-stress clinical environments (Aminihajibashi et al., 2022). Finally, self-care-focused trainings specifically designed for trauma-exposed clinicians demonstrate moderate reductions in secondary traumatic stress and burnout, with benefits sustained at follow-up when participants continue practicing skills introduced during training (Brillon et al., 2023; Deblinger et al., 2024; Halamová et al., 2022).

In addition to the literature on continuing education, an established body of research supports the role of clinical supervision in promoting provider well-being. Participation in clinical supervision is associated with lower levels of burnout and emotional exhaustion, as well as higher job satisfaction and staff retention (Hiebler-Ragger et al., 2021; Knudsen et al., 2008; Martin et al., 2021; Sellers et al., 2025). The quality of the supervisory relationship appears to be an especially important feature of clinical supervision. Strong supervisory alliances are linked to lower burnout and improved psychological well-being among providers (Hiebler-Ragger et al., 2021).

Supervision may exert its protective effects through multiple pathways, including enhancing providers’ sense of support and organizational commitment, increasing perceptions of workplace autonomy, empowerment, and fairness, and providing space to process challenging clinical encounters (Knudsen et al., 2008, 2013). Factors that strengthen these effects include regular and sufficient supervision, opportunities for reflective discussion, and a collaborative supervisory relationship characterized by trust and openness (Collins et al., 2025; Posselt et al., 2020; Sellers et al., 2025). However, not all supervision is beneficial; poorly structured or purely administrative supervision may fail to mitigate, and in some cases may even contribute to, provider stress, underscoring the importance of supervision quality (Martin et al., 2021, 2022).

## Reflective supervision to mitigate stress and enhance well-being

Reflective supervision is a model of clinical supervision that originated in child and family services and has since been adapted for use in other practice areas, including trauma treatment and grief counseling (Eggbeer et al., 2007; Ravalier et al., 2023; Vîşcu & Rad, 2024). A reflective approach to supervision centers the relational and emotional aspects of clinical practice by creating space for providers to examine how their experiences and reactions impact client care (Dawson & Chunga, 2023). Reflective supervision departs from traditional models of clinical supervision employed in substance use treatment settings, which often focus on crisis management, administrative and procedural oversight, and ethical and legal practice issues (Bride et al., 2009; Center for Substance Abuse Treatment, 2009; Ewer et al., 2015). Instead, reflective supervision emphasizes the supervisory relationship, the supervisee’s emotional responses to the work, and the integration of trauma-informed practices.

Research supports reflective supervision as a tool to improve both clinical competencies and provider well-being across behavioral health settings. Qualitative and mixed methods research in this area highlights that participation in reflective supervision reduces stress and enhances providers’ perceptions of organizational support and job satisfaction (Frosch et al., 2019; Graham & Killick, 2019; Spielberger et al., 2022; Susman-Stillman et al., 2020; Tobin et al., 2024). In interviews, providers also perceive increased insight and emotional processing following reflective supervision (Barron et al., 2022; Dempsey et al., 2008; Huffhines et al., 2023). Quantitative findings link participation in reflective supervision to greater resilience, improved self-efficacy and capacity for reflection, and reductions in burnout (Eaves et al., 2022; Frosch et al., 2018; Shea et al., 2022; Spielberger et al., 2022). Though evidence is limited, longitudinal research suggests that sustained engagement in reflective supervision can enhance providers’ capacity to manage work-related stress (Frosch et al., 2018). At the organizational level, settings that incorporate reflective and relational supervision practices have reported improved staff retention and service quality (Martin et al., 2021; Sellers et al., 2025). Taken together, these findings underscore reflective supervision as a promising, if underutilized, strategy for strengthening workforce resilience and enhancing quality of care in high-intensity clinical environments.

## The current study

This study sought to characterize current supervision and training practices in OTPs and to assess their association with VT symptoms and burnout among clinical staff. Specifically, we aimed to: (1) provide a preliminary understanding of the OTP supervision and training landscape by documenting the availability of clinical supervision and training related to VT and burnout in a national sample of OTPs; (2) assess receptivity to a reflective supervision program tailored to the OTP environment, including the likelihood that clinics would adopt such a program and that clinical staff would participate; and (3) examine associations among availability of clinic-level training, participation in clinical supervision, and staff VT symptoms and burnout. We hypothesized that both clinic-level training availability and participation in clinical supervision would be associated with lower VT symptoms and burnout, but that clinical supervision may have stronger effects. Clarifying the relationships among training, clinical supervision, and staff trauma-related functioning has the potential to inform the conceptualization and development of targeted interventions to support the OTP workforce and, ultimately, improve the quality and continuity of care for individuals with opioid use disorder.

## Method

### Participants and procedures

Data for this study were drawn from a cross-sectional, national survey of OTP administrators and staff conducted between June and August 2024. Details of the parent study methodology have been reported previously (Meyerson et al., 2025). Briefly, a stratified national sample of 1,000 OTPs was constructed using the SAMHSA directory of OTP clinics, with stratification based on state policy context and county-level opioid overdose mortality rates. Study staff contacted 646 OTPs by telephone. Of these, 138 clinic representatives agreed to receive study information and provided contact details for a clinic administrator. OTP administrators who completed the 50-item online survey were offered a $30 electronic gift card.

Staff participants were recruited from clinics whose administrators completed the parent survey. Administrators were asked to display a recruitment flyer in staff-only clinic areas. The flyer included a QR code and URL linking to a study information page where staff could review study details and provide informed consent electronically. Eligible staff were required to be at least 18 years of age, employed full time at a participating OTP clinic, and working in one of the following roles: medical (e.g., physician, physician assistant, nurse), counseling (e.g., counselor, clinical supervisor), or other staff roles (e.g., peer support, front desk, administrative staff, case management, patient navigation, outreach). Eligible staff who agreed to participate completed an online survey hosted on the secure Qualtrics platform assessing workplace experiences (e.g., years of work experience, current and prior roles) and functioning including VT and burnout. Staff received a $40 electronic gift card upon completion. Each participating clinic was assigned a unique four-digit identification code that linked administrator and staff survey responses from the same clinic. The study protocol was approved by the University of Arizona Human Subjects Protection Program. The survey instrument, data and data dictionary can be accessed via the National Addiction & HIV Data Archive Program (NAHDAP) through Inter-University Consortium for Political and Social Research (NAHDAP-228330) per the HEAL data ecosystem data sharing requirements.

### Measures

#### Training in vicarious trauma and burnout

At the clinic level, administrator respondents reported on trainings available and/or required of staff at their clinic. For both vicarious/work-related trauma and managing burnout, administrators selected whether each training type was offered to staff, required of staff, or not offered or required at their clinic.

#### Clinical supervision

Counseling staff were asked to report on their participation in clinical supervision by responding to the prompt: *‘Clinical supervision is a formal process of professional support, reflection, and learning in which a more experienced counselor/clinician provides case consultation and professional development to a less experienced counselor/clinician. The purpose of clinical supervision is to support competent, high-quality care and improve patient outcomes. Do you currently receive clinical supervision?’* Response options included: *‘Yes, at my workplace’; ‘Yes, but not at my workplace’;* and *‘No.’* Counselors who endorsed participation in clinical supervision responded to follow-up questions capturing clinical supervision frequency on a Likert scale from 1 (*a few times a year*) to 5 (*weekly or more*) and satisfaction with current clinical supervision on a Likert scale from 1 (*totally dissatisfied*) to 6 (*totally satisfied*). Further, clinic administrators reported on their willingness to implement a program of reflective coaching/supervision in their clinics, and frontline clinical staff, including counselors, peer support workers, and case managers, reported the likelihood that they would participate if this service was offered in their clinics.

#### Vicarious trauma

Staff VT was measured using the eight-item Vicarious Trauma Scale (VTS; Vrklevski & Franklin, 2008), which assesses exposure to trauma through clinical work and associated emotional and cognitive responses. Items were rated on a seven-point Likert scale ranging from 1 (*strongly disagree*) to 7 (*strongly agree*). The first two items assessed VT exposure at work (*‘My job involves exposure to distressing materials and experiences’; ‘My job requires exposure to traumatized or distressed clients’*), and the remaining items measured the severity of affective and cognitive VT symptoms. As recommended in prior research (Jimenez et al., 2021), we used a composite VTS symptom score, excluding exposure items, in analyses (*α* = .78) The VTS has demonstrated reliable and valid psychometric properties in prior studies (Aparicio et al., 2013; Benuto et al., 2018).

#### Burnout

Clinic administrators reported on their perceptions of staff burnout on an investigator-created single-item measure (‘In your opinion, how much is staff burnout an issue in your clinic?’). Response choices ranged from 1 (*not an issue*) to 4 (*a big issue*). Staff burnout was assessed using a validated single-item measure (Dolan et al., 2015). In response to the prompt, *‘Job burnout is a type of stress linked to work. It includes feeling worn out physically or emotionally and may also involve feeling ineffective or powerless. Overall, based on your definition of burnout, how would you rate your level of burnout?’* Staff rated their level of burnout among five response options. The first two indicated the absence of burnout: *‘1) I enjoy my work. I have no symptoms of burnout’;* and *‘2) Occasionally I am under stress, and I don’t always have as much energy as I once did, but I don’t feel burned out.’* The remaining three response options reflected escalating levels of burnout: *‘3) I am definitely burning out and have one or more symptoms of burnout, such as physical and emotional exhaustion’; ‘4) The symptoms of burnout that I’m experiencing won’t go away. I think about frustrations at work a lot’;* and *‘5) I feel completely burned out and often wonder if I can go on. I am at the point where I may need some changes or may need to seek some sort of help.’* A score of 3 or higher indicated clinically significant burnout symptoms. This measure has demonstrated convergent validity with longer validated measures of burnout (Trockel et al., 2018).

### Data preparation and analysis

Descriptive statistics characterized sample demographics, clinic-level training availability, acceptability of reflective supervision, and staff trauma-related functioning (i.e., VT and burnout). Continuous variables were examined to assess distributional properties and identify non-normality prior to model estimation (Afifi et al., 2007). Staff reports of burnout were moderately skewed (pre-transformation skew = 1.15) and were log transformed prior to regression analyses (post-transformation skew = −0.06). Intraclass correlation coefficients (ICCs) were estimated using random-intercept models to estimate the proportion of variance attributable to clinic-level clustering for study outcomes. ICC values for VT (ICC = .030) and burnout (ICC = .099) were small and varied modestly across model specifications, consistent with limited clustering and small cluster sizes. Corresponding design effects for VT (DEFF = 1.04) and burnout (DEFF = 1.19) were examined to assess the degree to which clustering inflated standard errors relative to a random sample of the same size with no data clusters. Design effects suggested only modest inflation of variance estimates due to clustering, with corresponding standard errors inflated by approximately 2% for VT and 9% for burnout.

To examine associations between training availability and staff outcomes, a series of linear regression models were estimated. Models evaluated the relationship between binary clinic-level variables (VT and burnout training availability) and standardized outcomes (staff VT symptoms and burnout) while controlling for demographic covariates, including participant age, years working in the SUD treatment field, and ethnicity (non-Hispanic white = 1). Models were run with the total staff sample (*N* = 83), and, in sensitivity analyses, with a subsample restricted to frontline clinical staff (i.e., counselors, case managers, peer support workers; *n* = 48). Additional regression models evaluated associations between participation in clinical supervision and VT and burnout outcomes among counseling staff only (i.e., those who reported on clinical supervision participation). For each effect, the regression coefficient (β) represents the mean difference in the outcome between the groups represented by the binary predictor (e.g., availability of training or clinical supervision) expressed in standard deviation units. Said another way, the β coefficient indicates how many standard deviations higher or lower the outcome is when training or supervision is available compared to when it is not available.

Due to the nested data structure, in all models, cluster-robust standard errors were estimated to account for non-independence of observations of staff within clinics. A bias-reduced cluster-robust variance estimator was applied, and hypothesis tests were conducted using saddlepoint approximations, which provide improved *p*-value accuracy under small cluster conditions. All regression models were conducted in RStudio v4.5.2 (R Core Team, 2025) using the *lm()* function for model estimation and the *lmtest* and *clubSandwich* packages to compute cluster-robust variance estimators and hypothesis tests. Adjusted *R*^2^ values evaluated the proportion of variance in the outcome explained within each regression model. Statistical significance was assessed at *p* < .05.

## Results

### Participant characteristics

Of the 138 OTP clinics that provided administrator contact information, 44 administrators (each representing a unique clinic) across 21 U.S. states completed the national clinic survey (32% clinic response rate; see Table 1). All administrators identified their clinic role as site supervisor or clinic manager/director. Staff members from 28 of the 44 clinics included in the national clinic survey participated in a staff survey. Of 86 OTP staff participating in the survey, 83 completed all items and were retained in the analytic sample (see Table 2). The staff sample included medical personnel (*n* = 24; 29%), counselors (*n* = 30; 36%), and other staff in non-medical and non-counseling roles (e.g., front desk staff, peer support workers, case managers, *n* = 29; 35%). The mean age of OTP staff was 44.3 years (range = 19–69), and the average length of employment in substance use disorder (SUD) treatment was 7 years (range = 0–20). Medical staff reported the greatest mean professional experience in methadone treatment settings (5.1 years). Counselors reported the greatest mean professional experience in general or nonspecific SUD treatment settings (8.2 years). Most respondents (81.9%) identified as female.

Approximately half of the staff sample (51.8%) indicated that their roles involved exposure to distressing materials and experiences, and 86.7% reported routine contact with clients who were traumatized or emotionally distressed. Despite high exposure to client trauma, variability in VT symptomatology was observed. Specifically, 36.1% of respondents reported minimal or no VT symptoms, 56.6% reported moderate symptoms, and 7.2% endorsed high symptom severity. Medical staff reported the highest mean VT symptom scores (*M* = 23.46, *SD* = 6.26), followed by counselors (*M* = 19.23, *SD* = 7.29) and other staff (*M* = 18.00, *SD* = 7.22). Overall, 20% of staff scored above the established cutoff for burnout. Counselors demonstrated the highest prevalence of burnout (30%), followed by medical staff (20.8%) and other personnel (10.3%). Burnout scores were positively and significantly correlated with VT symptoms across all staff groups (*r* = 0.41–0.65, *p* < .05).

### Availability of training and clinical supervision

According to clinic administrators, training in vicarious or work-related trauma was available to staff in 81.4% of clinics, and 41.9% of clinics required staff to participate in VT training (see Table 1). Training in managing burnout was even more widely available (90.7% of clinics), with approximately half of clinics (48.8%) requiring staff to complete this training. Just under half of administrators (47.7%) identified staff burnout as a concern within their clinics, with 38.6% describing it as ‘an issue’ and 9.1% as ‘a big issue.’ Administrator perception of staff burnout did not differ based on the availability of training in either managing burnout, *t*(41) = 0.562, *p* = .58, or VT, *t*(41) = 0.843, *p* = .40.

Most counseling staff (86.7%) reported receiving some form of clinical supervision, and most (83.3%) received supervision within their clinic, while only one reported working with an external supervisor. The frequency of clinical supervision participation varied across counselors in this sample: 56.7% reported receiving supervision at least weekly, 6.7% every two weeks, 13.3% monthly, and 10.0% every other month or less. Among counselors receiving clinical supervision, overall satisfaction was moderately high. About half (53.8%) reported they were ‘totally satisfied’ with clinical supervision, while 26.9% were satisfied, 11.5% somewhat satisfied, and 7.7% reported feeling totally dissatisfied with clinical supervision.

### Acceptability of reflective supervision

Most administrators (83.3%) indicated that they would be likely to implement reflective coaching/supervision for clinical staff. Nearly half (46.5%) reported a willingness to adopt reflective coaching/supervision delivered by a licensed, trained supervisor without restrictions. An additional 37.3% of administrators indicated willingness to implement reflective coaching/supervision on the condition that the clinical supervisor was staff in their clinic or clinic system. Among the 48 frontline clinical staff included in the sample, 89.6% expressed interest in participating in reflective coaching/supervision, with 54.2% indicating they would be likely to participate and 35.4% indicating they would be highly likely to participate.

### Associations among staff training, clinical supervision, and trauma-related functioning

Trauma-related functioning among OTP staff, including VT symptoms and burnout, differed by the availability of clinic-level training (see Table 3). Specifically, the availability of workplace training on VT was associated with significantly *higher* VT symptom scores among all staff (β = 0.70, 95% CI [0.44, 0.96], *p* < .001), indicating that staff in clinics where training was available scored 0.70 *SD* higher on average on the VT symptom scale as compared to staff in clinics where training was not offered. This effect was comparable in the subset of frontline clinical staff (β = 0.66, 95% CI [0.22, 1.10], *p* = .02). Similarly, when clinics offered training in VT, staff in the total sample reported significantly elevated burnout (β = 0.46, 95% CI [0.10, 0.82], *p* = .04; i.e., 0.46 *SD* higher on average than staff in clinics where training was not available). This effect was replicated in the subset of frontline clinical staff (β = 0.61, 95% CI [0.12, 1.09], *p* = .04).

The same pattern of results was observed when training on managing burnout was available to staff. In the total sample, availability of clinic-level burnout training was associated with higher staff VT symptoms (β = 0.63, 95% CI [0.29, 0.97], *p* = .008) and higher burnout (β = 0.55, 95% CI [0.12, 0.97], *p* = .04). Again, the findings were similar in the subset of frontline clinical staff. When clinics offered training on managing burnout, staff VT symptoms (β = 0.58, 95% CI [0.07, 1.09], *p* = .04) and burnout scores (β = 0.66, 95% CI [0.02, 1.29], *p* = .05) were elevated. Models explained a modest proportion of variance in VT symptoms and burnout (adjusted *R*^2^ = 0.05–0.12; see Table 2).

Notably, a different pattern of findings was observed when counseling staff participated in clinical supervision (see Table 4). Participation in clinical supervision was associated with *lower* VT symptoms (β = −1.38, 95% CI [−1.92, −0.83], *p* = .001; i.e., 1.38 *SD* lower on average as compared to counselors who did not participate in supervision) and *lower* burnout (β = −1.21, 95% CI [−2.08, −0.33], *p* = .03; i.e., 1.21 *SD* lower on average as compared to counselors who did not participate in supervision). Models explained a moderate proportion of the variance in counselors’ VT symptoms (29%) and burnout scores (18%).

## Discussion

This study provides a preliminary examination of clinical supervision, training practices, and trauma-related functioning among a diverse multistate sample of OTP staff. Survey findings from OTP administrators and staff revealed three key themes. First, staff experienced high exposure to client trauma, alongside meaningful variability in VT symptoms and burnout. Second, availability of clinic-level training on VT and managing burnout was common, but training availability was not associated with better staff outcomes. Third, the results, while preliminary, indicate a potentially protective role of clinical supervision for mitigating VT symptoms and burnout among counseling staff. This study contributes to a limited but growing literature on workforce well-being and resilience in OTP settings (Coffee et al., 2025; Linde-Krieger et al., 2025). The findings have implications for future research and practice, including the potential to inform approaches to training and clinical supervision to mitigate provider stress.

### OTP staff VT and burnout

In this study, OTP staff across medical, counseling, and other roles reported substantial exposure to client trauma in their daily work, with the vast majority indicating regular contact with distressed or traumatized clients. Despite high exposure to secondary trauma, VT symptoms varied considerably across staff, suggesting that individual, organizational, or contextual factors shape and mitigate how staff experience and manage work-related trauma (Ewer et al., 2015; Gkremou et al., 2026; Guitar & Molinaro, 2017). Burnout was also present in a sizable minority of staff (20%), particularly among counselors (30%), and was moderately correlated with VT symptoms, consistent with prior literature linking secondary trauma to occupational stress and compassion fatigue (Cummings et al., 2021; Gkremou et al., 2026; Klingemann et al., 2024). The prevalence of clinical VT symptoms and burnout in this sample was consistent with prior studies of social workers, therapists, and other mental health professionals (Bride, 2007; Kelly et al., 2026; Kok et al., 2016; Morse et al., 2012; O’Connor et al., 2018). Across SUD and other treatment fields, VT and burnout are linked with reduced job satisfaction and elevated staff turnover (Guitar & Molinaro, 2017; Raunick et al., 2015; Showalter, 2010), particularly for frontline clinical workers including social workers and counselors (Bride & Kintzle, 2011; Canfield, 2005; O’Connor et al., 2018; Volker et al., 2010). OTP staff in counseling roles may be especially vulnerable to the negative effects of VT, given high caseloads, limited clinic resources, and the emotionally taxing work of supporting clients through crisis, treatment interruption, and overdose. The high prevalence of staff stress observed in this sample suggests a need to expand organizational supports for frontline workers in high-intensity treatment settings such as OTPs (e.g., adequate staffing to reduce workloads, regular mentoring/supervision, confidential screening and navigation to trauma services, protected time for documentation/administrative activities, flexible scheduling, leave time/paid time off).

### Training and trauma-related functioning

Behavioral health and SUD treatment clinics commonly offer staff training for professional development and as a resource to support staff well-being. Extant studies suggest that training is necessary and efficacious for developing professional competencies, acquiring field specific skills, and learning new treatment techniques or modalities (Frank et al., 2020; Valenstein-Mah et al., 2020). However, evidence for the effectiveness of staff training to reduce stress and improve wellness is less robust, with most studies indicating that improvements are achieved and maintained when training is evidence-based, interactive, and ongoing (e.g., coaching; Collett et al., 2026; Garcia et al., 2026; Mösler et al., 2023; Workman et al., 2026). In the present study, training availability was not associated with lower VT or burnout; instead, it was consistently associated with higher staff-reported stress. This unexpected pattern should not be interpreted as evidence that training increases VT or burnout, but rather it may indicate that currently available training practices are insufficient as standalone supports; that training increases staff awareness and reporting of distress; or that training availability marks clinics with greater underlying stress burden.

The present findings may reflect a disconnect between training format, content, frequency, and/or intensity and the needs of staff in high-stress work environments. When delivered as a one-time or didactic intervention, training is likely inadequate to mitigate the ongoing emotional demands on a trauma-exposed workforce. Further, training in work-place trauma and its consequences potentially raises staff members’ awareness of distress, including VT and burnout, without offering practical coping strategies or ongoing support. It is also possible that positive associations of training availability and staff VT symptoms and burnout may be explained by underlying differences in clinic environments. For example, it may be that clinics offering these trainings provided a safer space for staff disclosure of VT symptoms and burnout. Indeed, when staff experience greater distress, clinics may be more likely to implement training initiatives in response to identified needs. In this sense, training availability may function as a proxy for higher-risk or higher-burden clinical settings rather than an intervention effect.

These explanations are not mutually exclusive and cannot be disentangled in the current cross-sectional study. We did, however, assess concurrent links among administrator ratings of staff burnout, staff self-reports of burnout, and training availability. Results suggest that (1) administrator perceptions of staff burnout were not associated with training availability and did not appear to align with staff self-reports, and (2) there was no evidence that administrator perception of greater staff burnout was associated with greater availability of training in VT or managing burnout. In other words, administrators may perceive staff burnout differently than staff perceive their own burnout, and training did not appear to be systematically implemented, uniquely tailored, or curated in response to perceived levels of staff distress. Taken together, these findings suggest training availability alone may be an imprecise indicator of organizational responsiveness to addressing workforce stress.

### Clinical supervision and trauma-related functioning

In contrast to training availability, participation in clinical supervision was associated with significantly *lower* VT symptoms and burnout among counseling staff. These findings should be interpreted cautiously given the small counseling subsample and the limited number of counselors not receiving supervision. They are, however, consistent with the possibility that clinical supervision may be protective in OTP settings. Clinical supervision, particularly when supportive or reflective in nature, may provide opportunities for staff to process difficult emotions that arise in clinical work (Dawson & Chunga, 2023; Eggbeer et al., 2007; Vîşcu & Rad, 2024). In contrast to training, clinical supervision represents an ongoing, interpersonal form of support, which may support coping, adaptation, and resilience and, therefore, be better aligned with the chronic and cumulative nature of trauma exposure in OTP settings. Relational and trauma-informed supervision approaches can also support empathy and clinical skill development while validating the effects of secondary trauma exposure – elements that are not typically addressed through standard training approaches (Berger & Quiros, 2014; Dawson & Chunga, 2023; Miller & Sprang, 2017; Varghese et al., 2018). The observed associations are consistent with theoretical models of trauma-informed care and workforce sustainability, which emphasize ongoing relational support as a key mechanism for mitigating the impact of chronic exposure to client trauma. Notably, most counselors in this sample reported receiving supervision, and satisfaction with supervision was generally high, indicating that clinical supervision is likely feasible and acceptable within OTP settings.

Relatedly, both administrators and staff expressed strong interest in implementing or participating in reflective supervision or coaching models. This pattern suggests an opportunity to implement workforce interventions beyond traditional training formats and administrative supervision approaches. Future research should examine trauma-informed reflective supervision models tailored to OTP settings, including their feasibility, implementation barriers, and impact on both staff well-being and quality of care.

### Limitations

Several limitations should be considered when interpreting these findings. First, the cross-sectional design of this study precludes causal inference. The observed associations between training availability and higher staff VT symptoms and burnout are preliminary and may reflect unmeasured contextual differences rather than causal or directional relationships. Future research should seek to replicate these findings in prospective studies that can assess temporal ordering and potential mechanisms (e.g., training format, frequency, intensity, uptake). Second, although the sample was geographically diverse, the sample size was modest, particularly within subgroups such as counseling staff, and cluster sizes within clinics were small. These features may limit precision of estimates and reduce the general-izability and stability of the findings. Third, study participation was voluntary at both the clinic and staff levels, introducing the possibility of self-selection and reporting biases. Fourth, measures of training availability were reported by administrators and did not capture staff training uptake or the content, quality, or intensity of training received by staff. Therefore, we were not able to evaluate specific features of training that may attenuate staff outcomes. Finally, although our survey assessed the location (internal or external to their workplace) and frequency of counselors’ participation in clinical supervision as well as satisfaction with supervision, it did not assess other potentially important features, such as supervision quality or focus (e.g., administrative vs. reflective; Collins et al., 2025; Hiebler-Ragger et al., 2021; Posselt et al., 2020; Sellers et al., 2025).

### Clinical and research implications

Sustaining a stable and effective substance use treatment workforce requires attention to both practice quality and innovation and the emotional demands placed on treatment providers. Consistent with prior research (Collett et al., 2026; Gkremou et al., 2026), the current findings suggest that training as a standalone strategy for addressing VT reactions and burnout may be insufficient. In OTP settings, where staff routinely engage with clients experiencing chronic and complex trauma, workforce interventions may be most effective when they include opportunities for emotional processing, reflection, and ongoing skill development (Berger & Quiros, 2014; Gkremou et al., 2026; Mavridoglou et al., 2026). These findings therefore point toward the potential value of interactive, practice-based professional development approaches that are integrated with ongoing supervision rather than offered as isolated trainings.

Clinical supervision, particularly when grounded in reflective and trauma-informed principles, may be a promising strategy to support staff well-being in OTPs (El Amanni & Bartley, 2025; Knudsen et al., 2013; Martin et al., 2021). Findings from this study suggest that both administrators and clinical staff find reflective supervision to be an acceptable professional development tool for the OTP setting. What is not yet clear is how reflective supervision may be best tailored to the needs, experiences, and varying clinical and professional backgrounds of OTP providers.

The associations between supervision and lower VT symptoms and burnout observed here should be viewed as hypothesis-generating, rather than mechanistic. Prior work suggests that supervision may serve as a mechanism for mitigating the cumulative effects of secondary trauma exposure (Hiebler-Ragger et al., 2021; Knudsen et al., 2008, 2013). Features of clinical supervision, including the format, focus (e.g., administrative vs. reflective), and quality of the supervisory relationship, as well as clinic context (e.g., leadership buy-in, resource availability) may modify the impact of clinical supervision on provider well-being and quality of care (Collins et al., 2025; Hiebler-Ragger et al., 2021; Posselt et al., 2020; Sellers et al., 2025). In this context, the present findings support further study of structured, high-quality supervision models in OTPs that prioritize relational engagement, emotional processing, and reflective practice (Bell et al., 2024; Martin et al., 2021; Morelen et al., 2022). Unlike traditional models of supervision in SUD treatment settings that are available to masters-level counselors, programs of reflective, trauma-informed supervision designed for the OTP setting could serve as a protective factor for staff across multiple client-facing roles, including counselors, case managers, treatment navigators, and peer support workers. Implementation efforts would benefit from careful consideration of supervision quality, consistency, and alignment with the needs of staff working in high-intensity treatment environments (Aristy, 2025).

The current findings also offer important directions for future research. Extant evidence on the needs of OTP staff is extremely limited, despite high levels of stress, burnout, and turnover (Beitel et al., 2018; Coffee et al., 2025; Murphy & Kruis, 2023). Specifically, there is a need for more rigorous evaluations of workforce interventions in OTP settings. Future studies should employ longitudinal and experimental designs to clarify temporal relationships and test whether training and supervision influence OTP staff outcomes. In particular, research should aim to disentangle the effects of training content, format, and intensity, as well as to examine how reflective and trauma-informed supervision models can be adapted and sustained in OTP contexts (El Amanni & Bartley, 2025; Morelen et al., 2022). Implementation science frameworks may be useful for identifying organizational facilitators and barriers to adoption, including leadership support, workforce capacity, and regulatory constraints. Additionally, qualitative methods can be used to better understand provider needs and experiences. Such work should include a range of staff roles, particularly paraprofessional and peer support workers whose contributions to SUD treatment are vital but understudied (Bell et al., 2024).

## Conclusion

In sum, training related to VT and burnout was widely available across participating OTPs, but training availability was not associated with better staff outcomes in this cross-sectional study. Clinical supervision, particularly approaches that emphasize reflection, emotional processing, and relational support, appears to be a feasible and acceptable workforce support strategy in OTPs and merits further study. Moving beyond didactic training and administrative models of supervision represents a paradigm shift in addiction treatment. Future longitudinal and experimental research is needed to identify optimal implementation strategies to promote staff resilience in high-stress treatment environments. Ultimately, this work has the potential to inform more effective approaches to training and supervision that reduce provider stress, prevent burnout and turnover, and improve the quality and continuity of care in OTP settings.

## Figures and Tables

**Table 1.** OTP clinic and administrator characteristics (*N* = 44).

	Clinics/Administrators
Clinic Location/U.S. Region N (%)	
Northeast: VT, RI, NY, PA	6 (13.6%)
Midwest: IN, IL, MN	10 (22.7%)
South: NC, GA	13 (29.5%)
West: CO, NM, AZ, NV, WA, OR, CA	15 (34.1%)
Community Type N (%)	
Urban	37 (84.1%)
Rural	7 (15.9%)
# of patients served *Mean (SD)*	286.0 (297.6)
# of staff *Mean (SD)*	15.50 (11.16)
Staff to patient ratio *Mean (SD)*	0.17 (0.63)
Is staff burnout an issue at your clinic? N (%)	
Not an issue	4 (9.1%)
Not much of an issue	19 (43.2%)
An issue	17 (38.6%)
A big issue	4 (9.1%)
Staff training on managing burnout N (%)	
Offered (not required)	18 (41.9%)
Required	21 (48.8%)
Not offered or required	4 (9.3%)
Staff training on vicarious/work-related trauma N (%)	
Offered (not required)	17 (39.5%)
Required	18 (41.9%)
Not offered or required	8 (18.6%)
Likelihood of implementing reflective supervision tailored to OTPs	
Likely to implement	35 (83.3%)
Unlikely to implement	7 (16.7%)
Under what conditions would your clinic implement reflective supervision?	
Willing without limitations	20 (46.5%)
Willing if delivered by a supervisor employed at clinic or company	16 (37.3%)
Willing under another condition	7 (16.3%)

**Table 2.** OTP staff demographics stratified by clinic role (*N* = 83).

	All Staff	Medical	Counselor	Other Staff
# of participants *N (% of total sample)*	83 (100%)	24 (29%)	30 (36%)	29 (35%)
Age *Mean (SD)*	44.3	41.2 (8.94)	47.9 (11.79)	43 (11.5)
Race N (%)				
White	51 (61.4%)	17 (70.8%)	16 (53.3%)	18 (62.1%)
Black	21 (25.3%)	4 (16.7%)	11 (36.7%)	6 (20.7%)
American Indian/Native American or Alaska Native	3 (0.04%)	1 (4.2%)	0 (0%)	2 (6.9%)
Asian	1 (0.01%)	0 (0%)	1 (3.3%)	0 (0%)
Native Hawaiian or Other Pacific Islander	1 (0.01%)	1 (4.2%)	0 (0%)	0 (0%)
Other	9 (10.8%)	3 (12.5%)	2 (6.7%)	4 (13.8%)
Ethnicity N (%)				
Hispanic/Latine	17 (20.5%)	6 (25%)	2 (6.7%)	9 (31.0%)
Non-Hispanic/Latine	66 (79.5%)	18 (75%)	28 (93.3%)	20 (69.0%)
Gender				
Cisgender Female	68 (81.9%)	20 (83.3%)	24 (80%)	24 (82.8%)
Cisgender Male	10 (12.0%)	1 (4.2%)	5 (16.7%)	4 (13.8%)
Gender Nonbinary	2 (0.02%)	2 (8.3%)	0 (0%)	0 (0%)
Other (please enter)	3 (0.04%)	1 (4.2%)	1 (3.3%)	1 (3.4%)
U.S. Regions N (%)				
Northeast: VT, RI, NY, PA	12 (14.5%)	5 (20.8%)	2 (6.7%)	5 (17.2%)
Midwest: IN, IL, MN	23 (27.7%)	4 (16.7%)	10 (33.3%)	9 (31.0%)
South: NC,GA	20 (24.1%)	6 (25.0%)	9 (30%)	5 (17.2%)
West: CO, NM, AZ, NV, WA, OR, CA	28 (33.7%)	9 (37.5%)	9 (30%)	10 (34.5%)
# of years worked at clinic *Mean (SD)*	3.90 (3.80)	4.04 (3.79)	3.1 (3.26)	4.41 (4.26)
# of years worked in methadone treatment *Mean (SD)*	4.48 (4.73)	5.13 (4.56)	4.3 (4.94)	3.93 (4.72)
# of years worked in SUD treatment *Mean (SD)*	7.20 (5.70)	6.79 (6.40)	8.17 (5.53)	6.28 (5.34)
Burnout score *Mean (SD)*	2.02 (0.88)	2.21 (1.10)	2.13 (0.86)	1.76 (0.64)
Vicarious trauma symptoms *Mean (SD)*	20.02 (7.26)	23.46 (6.26)	19.23 (7.29)	18.00 (7.22)
Clinical supervision frequency N (%)				
Every other month or less			3 (10.0%)	
About every month			4 (13.3%)	
About every two weeks			2 (6.7%)	
Weekly or more			17 (56.7%)	
Satisfaction with clinical supervision *Mean (SD)*			5.12 (1.40)	

**Table 3.** Linear regression models predicting OTP staff vicarious trauma symptoms and burnout.

	All OTP Staff (N = 83across 28 clinics)								Frontline Clinical Staff (N = 48across 23 clinics)							
	VT Symptoms				Burnout				VT Symptoms				Burnout			
	Cluster Robust				Cluster Robust				Cluster Robust				Cluster Robust			
	β	95% CI			β	95% CI			β	95% CI			β	95% CI		
Predictor	*(SE)*	*LL*	*UL*	*p*	*(SE)*	*LL*	*UL*	*p*	*(SE)*	*LL*	*UL*	*p*	*(SE)*	*LL*	*UL*	*p*
VT training available (1 = yes)	0.70 (0.12)	0.44	0.96	<.001	0.46 (0.17)	0.10	0.82	.04	0.66 (0.20)	0.22	1.10	.02	0.61 (0.22)	0.12	1.09	.04
Adjusted R^2^	0.12				0.08				0.09				0.10			
Burnout training available (1 = yes)	0.63 (0.16)	0.29	0.97	.008	0.55 (0.20)	0.12	0.97	.04	0.58 (0.24)	0.07	1.09	0.04	0.66 (0.29)	0.02	1.29	.05
Adjusted R^2^	0.08				0.08				0.05				0.09			

Note: VT = vicarious trauma. SE = cluster-robust standard error, clustered by clinic; LL = lower limit of 95% confidence interval; UL = upper limit of 95% confidence interval. Models adjusted for participant age, years working in the SUD treatment field, and ethnicity (non-Hispanic white = 1).

**Table 4.** Linear regression models with cluster-robust standard errors predicting vicarious trauma symptoms and burnout among counseling staff (*N* = 30 across 21 clinics).

	VT Symptoms				Burnout			
Predictor	Cluster Robust				Cluster Robust			
	β	95% CI			β	95% CI		
	*(SE)*	*LL*	*UL*	*p*	*(SE)*	*LL*	*UL*	*p*
Receiving clinical supervision (1 = yes)	−1.38 (0.24)	−1.92	−0.83	.001	−1.21 (0.39)	−2.08	−0.33	.03
**Adjusted R^2^**	0.29				0.18			

Note: VT = vicarious trauma. SE = cluster-robust standard error, clustered by clinic; LL = lower limit of 95% confidence interval; UL = upper limit of 95% confidence interval. Models adjusted for participant age, years working in the SUD treatment field, and ethnicity (non-Hispanic white = 1).
